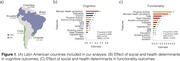# Socioeconomic and health‐related disparities associated with healthy brain aging in Latin American countries

**DOI:** 10.1002/alz.093131

**Published:** 2025-01-09

**Authors:** Lucas Uglione Da Ros, Wyllians Vendramini Borelli, Marco Antônio de Bastiani, Cristiano Schaffer Aguzzoli, Lucas Porcello Schilling, Hernando A Santamaría‐García, Tharick A. Pascoal, Pedro Rosa‐Neto, Jaderson Costa da Costa, Agustín Ibáñez, Claudia Kimie Suemoto, Eduardo R. Zimmer

**Affiliations:** ^1^ Universidade Federal Do Rio Grade Do Sul, Porto Alegre Brazil; ^2^ Memory Center, Hospital Moinhos de Vento, Porto Alegre, RS Brazil; ^3^ Universidade Federal do Rio Grande do Sul, Porto Alegre, Rio Grande do Sul Brazil; ^4^ Brain Institute of Rio Grande do Sul, PUCRS, Porto Alegre, RS Brazil; ^5^ Pontificia Universidad Javeriana, Bogota Colombia; ^6^ University of Pittsburgh, Pittsburgh, PA USA; ^7^ McGill University, Montreal, QC Canada; ^8^ Brain Institute of Rio Grande do Sul ‐ Pontifícia Universidade Católica do Rio Grande do Sul, Porto Alegre, Rio Grande do Sul Brazil; ^9^ Global Brain Health Institute, Trinity College Dublin, Dublin Ireland; ^10^ Physiopathology in Aging Laboratory (LIM‐22), University of São Paulo Medical School, São Paulo, São Paulo Brazil; ^11^ Universidade Federal do Rio Grande do Sul, Porto Alegre Brazil

## Abstract

**Background:**

Latin American Countries (LACs) have major health‐related inequities due to historical, cultural, and social aspects. These factors have been suggested as important determinants of healthy aging in LACs. Here, we evaluated classic and socioeconomic risk factors for healthy brain aging across five large cohorts of LACs.

**Method:**

Risk factors for healthy aging were evaluated using machine‐learning models in 41,092 individuals across five LACs ([Brazil, n = 9,412], Colombia [n = 23,694], Chile [n = 1,301], Ecuador [n = 5,235], and Uruguay [n = 1,450] (**Fig. 1A**). Healthy brain aging was evaluated using z‐scored cognitive and functional ability data with selected risk factors (Age, Sex, Diabetes, Education, Isolation, House Condition, Hypertension, Heart Disease, Alcohol Consumption, Physical Activity, Smoking, Falls, and Mental Health Problems). The fitness of models was evaluated with Mean Standard Error (MSE) and Raw Mean Standard Error (RMSE) extracted from Ridge Regressions Models (adjusted p<0.05).

**Result:**

Regarding cognition, our machine‐learning model with LACs was significant. The most important risk factors were mental health symptoms, education, country, physical activity, alcohol consumption, falls, socioeconomic status, isolation, age, and smoking status. No significant effects of heart disease, hypertension, sex, and diabetes were found (**Fig. 1B**). The model assessing functionality in LACs was also significant and presented the following order of risk factors: physical activity, mental health symptoms, falls, heart disease, alcohol consumption, diabetes, sex, hypertension, age, SES, education, education, and smoking status. Country and isolation did not reach statistical significance (**Fig. 1C**).

**Conclusion:**

Our findings demonstrated that social and health disparities outweigh classic risk factors, such as age and sex, for cognitive and functional decline in LACs, highlighting the need to identify risk factors for healthy brain aging in underrepresented populations.